# Predictive value and accuracy of [^18^F]FDG PET/CT modified response criteria for checkpoint immunotherapy in patients with advanced melanoma

**DOI:** 10.1007/s00259-023-06247-8

**Published:** 2023-05-04

**Authors:** Narjess Ayati, Zahra Jamshidi-Araghi, Magdalena Hoellwerth, Gregor Schweighofer-Zwink, Wolfgang Hitzl, Peter Koelblinger, Christian Pirich, Mohsen Beheshti

**Affiliations:** 1 Cancer Imaging, Peter MacCallum Cancer Centre, Melbourne, Victoria Australia; 2 Division of Molecular Imaging and Theranostics, Department of Nuclear Medicine, University Hospital Salzburg, Paracelsus Medical University, Muellner Hauptstrasse 48, 5020 Salzburg, Austria; 3 Department of Nuclear Medicine, Shahid Rajaie Cardiovascular, Medical & Research Center, Tehran, Iran; 4 Department of Dermatology, University Hospital Salzburg, Paracelsus Medical University, Salzburg, Austria; 5 Biostatistics and Publication of Clinical Trial Studies, Research and Innovation Management (RIM), Paracelsus Medical University, Salzburg, Austria; 6 Department of Ophthalmology and Optometry, Paracelsus Medical University, Salzburg, Austria; 7 Research Program Experimental Ophthalmology & Glaucoma Research, Paracelsus Medical University, Salzburg, Austria

**Keywords:** Melanoma, Immunotherapy, Immune checkpoint inhibitors, PERCIMT, PERCIST5, imPERCIST5

## Abstract

**Purpose:**

Immune checkpoint inhibitors (ICIs) are widely used in metastatic melanoma and dramatically alter the treatment of these patients. Given the high cost and potential toxicity, a reliable method for evaluating treatment response is needed. In this study, we assessed tumor response in patients with metastatic melanoma treated with ICIs using three modified response criteria: PET Response Evaluation Criteria for Immunotherapy (PERCIMT), PET Response Criteria in Solid Tumors for up to Five Lesions (PERCIST5), and immunotherapy-modified PET Response Criteria in Solid Tumors for up to Five Lesions (imPERCIST5).

**Methods:**

Ninety-one patients with non-resectable stage IV metastatic melanoma who received ICIs were retrospectively enrolled in this study. Each patient had two [^18^F]FDG PET/CT scans performed before and after ICI therapy. Responses at the follow-up scan were evaluated according to PERCIMT, PERCIST5, and imPERCIST5 criteria. Patients were classified into four groups: complete metabolic response (CMR), partial metabolic response (PMR), progressive metabolic disease (PMD), and stable metabolic disease (SMD). To assess the “disease control rate,” two groups have been defined based on each criterion: patients with CMR, PMR, and SMD as “disease-controlled group (i.e., responders)” and PMD as the “uncontrolled-disease group (i.e., non-responders)”. The correspondence between metabolic tumor response defined by these criteria and clinical outcome was assessed and compared.

**Results:**

The response and the disease control rates were 40.7% and 71.4%, 41.8% and 50.5%, and 54.9% and 74.7% based on the PERCIMT, PERCIST5, and imPERCIST5 criteria, respectively. PERCIMT and imPERCIST5 showed significantly different disease control rates from that of PERCIST5 (*P* < 0.001), whereas it was not significant between PERCIMT and imPERCIST5. Overall survival was significantly longer in the metabolic responder groups than in the non-responder groups based on PERCIMT and PERCIST5 criteria (PERCIMT: 2.48 versus 1.47 years, *P* = 0.003; PERCIST5: 2.57 versus 1.81 years. *P* = 0.017). However, according to imPERCIST5 criterion, this difference was not observed (*P* = 0.12).

**Conclusion:**

Although the appearance of new lesions can be secondary to an inflammatory response to ICIs and indicative of pseudoprogression, given the higher rate of true progression, the appearance of new lesions should be interpreted deliberately. Of the three assessed modified criteria, PERCIMT appear to provide more reliable metabolic response assessment that correlates strongly with overall patient survival.

**Supplementary Information:**

The online version contains supplementary material available at 10.1007/s00259-023-06247-8.

## Introduction

Melanoma is a common skin tumor with increasing incidence, rapid progression, and a high rate of metastasis [1–3]. In recent years, the profound understanding of the pathogenesis of metastatic melanoma and the importance of the anti-tumor immune response and regulatory mechanism has created new treatment options for melanoma patients, including systemic treatment with immune checkpoint inhibitors (ICIs), leading to improved survival in patients with advanced stages of the disease [4, 5]. According to the latest clinical practice guidelines for the treatment of melanoma from the National Comprehensive Cancer Network (NCCN) and the European Society for Medical Oncology (ESMO), antibodies against programmed death-1 (anti-PD1) alone or in combination with anti-cytotoxic T-lymphocyte antigen-4 (anti-CTLA4) antibodies are considered first-line treatment for unresectable stage III and IV melanoma patients [6–9].

Despite these dramatic advances, approximately 40–45% of patients do not respond to immunotherapy [1, 10]. In addition, the mechanism of action of these agents differs significantly from conventional cytotoxic approaches (stimulation of inflammation rather than direct lysis of tumor cells) [1, 11]. This difference in the mechanism of action between immunotherapy and cytotoxic chemotherapy should be considered when assessing response to immunotherapy [12, 13].

Different patterns of response to treatment with ICIs have been described [7]. Pseudoprogression is an unusual pattern of tumor response, defined as an increase in lesion size and/or metabolic activity and/or formation of new lesions followed by a response. Although pseudoprogression has only been reported in 10% of patients [14], a distinctive feature of immunotherapy compared to chemotherapy is the delayed response to treatment with persistent responses that may persist even after treatment is discontinued [2, 11, 14]. The ICIs also cause side effects that resemble autoimmune reactions and are referred to as immune-related adverse events (irAE), which can raise challenges in interpreting standard imaging techniques [15].

Given these atypical patterns of responses, the efficacy of conventional criteria such as Response Evaluation Criteria in Solid Tumors (RECIST) or PET Response Criteria in Solid Tumors (PERCIST) in the therapeutic evaluation of ICIs is limited [1, 5].

These challenges affect all imaging modalities of treatment response assessment. At least four modified criteria have already been proposed for morphologic imaging: immune-related Response Criteria (irRC) (2009) [16], immune-related RECIST (irRECIST) (2013) [8], immune RECIST (iRECIST) (2017) [12], and immune-modified RECIST (imRECIST) (2018) [17], all of which, despite some differences, require confirmation of tumor progression on follow-up and do not necessarily consider the appearance of new lesions as disease progression [10].

[^18^F]FDG PET/CT, in combination with brain MRI, is the preferred imaging strategy for staging metastatic melanoma [18] and is also considered a relatively reliable tool for immunotherapy monitoring, though, as, with other modalities, it has some limitations in assessing response to immunotherapy [3, 5, 9, 19].

Therefore, several modified scales have been recently proposed to assess response to immunotherapy by [^18^F]FDG PET/CT imaging, comprising Prediction of Response to Immune Checkpoint Inhibitor Therapy (PECRIT) (2017) [19], PET Response Evaluation Criteria for Immunotherapy (PERCIMT) (2018) [6, 13], immunotherapy-modified PERCIST for up to Five Lesions (imPERCIST5) (2019) [20], immune PERCIST (iPERCIST) (2019) [21], and PERCIST up to 5 lesions (PERCIST5) (2022) [22]. Among the above criteria, iPERCIST, like modified morphological criteria, implements the need to confirm progressive disease on follow-up imaging [23, 24]. However, PERCIMT, PERCIST5, and imPERCIST5 approaches rely mainly on metabolic changes, require no mandatory follow-up confirmation, and seem to provide a more clear statement regarding disease status after treatment with ICIs. Nonetheless, given recent developments of ICIs in the treatment of advanced melanoma, there is an unmet need to establish standard response criteria in both routines as well as research clinical trials that reliably predict the prognostic outcome.

In this study, we evaluated the predictive value of PERCIMT, PERCIST5, and imPERCIST5 criteria in response to immunotherapy on [^18^F]FDG PET/CT for progression-free survival (PFS) and overall survival (OS) of patients with advanced melanoma treated with ICIs.

## Materials and methods

### Patients

**Fig. 1:**
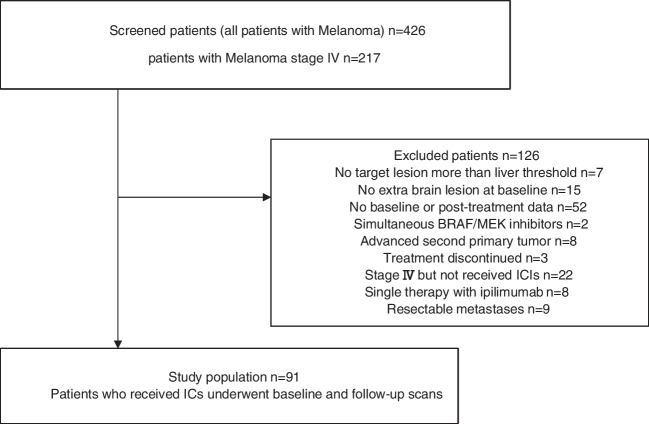
Diagram flowchart of patient selection process

In this retrospective study, clinical and imaging findings of 426 consecutive patients with advanced melanoma, who underwent baseline and follow-up [^18^F]FDG PET/CT between January 2015 and July 2021, were analyzed. Patients with stage IV metastatic melanoma treated with ICIs according to ESMO guidelines (Supplement 1) [25], including nivolumab or pembrolizumab or the combination with ipilimumab, and who had [^18^F]FDG PET/CT before and after at least two treatment sessions were included. The exclusion criteria were: metastases confined to the brain, concurrent other treatment [chemotherapy or B-Rapidly Accelerated Fibrosarcoma (BRAF) or MAP/ERK kinase (MEK) inhibitors], second cancer, resectable metastasis, and inadequate imaging parameters at baseline and/or therapy monitoring. A diagram flowchart summarizing the patient selection process is provided in Fig. 1. The patient’s demographic and clinical data were recorded and the effect of the ICI regimen and clinical risk factors, including age ≥ 75 years, gender, history of chemotherapy, BRAF mutation, history of treatment with BRAF/MEK inhibitors, and metastasis location, on the patient outcome were evaluated.

### Image acquisition and interpretation

Whole-body [^18^F]FDG PET/CT scans were performed based on a standard protocol [26]. Imaging was performed using two hybrid PET/CT scanners: Siemens 923/Biograph 64 mCT (Siemens Healthineers AG, Chicago, IL, USA) and Philips Ingenuity TF/Gemini TF 16 (Philips Medical Systems, Andover, MA, USA). Acquisition and reconstruction parameters were harmonized between scanners to minimize differences in standardized uptake values (SUV). Cross-calibration of the instrument is performed monthly at our center. Patients fasted for at least 4 h before intravenous [^18^F]FDG injection. An amount of 4 MBq/kg body weight for each patient was administered. The mean interval between [^18^F]FDG administration and the start of the acquisition was 60 ± 12 min. A non-contrast-enhanced low-dose CT scan (Siemens: Care Dose 4D, 100 kV, 59 mAs slice thickness 1.2 mm and pitch 1.5, Philips: 100 kV, 33 mAs, slice thickness 1.5 mm, and pitch 0.8) was performed for attenuation correction and precise anatomical location. Slice measuring 3-mm iterative algorithm (Siemens: PSF + TOF and Philips: BLOB-OS-TF) were used for reconstruction in both scanners. PET data were corrected to account for scattering, attenuation, random coincidence, and scanner dead time.

Both PET/CT scanners were EANM Research Ltd./European Association of Nuclear Medicine (EARL/EANM) accredited.

SUV normalized to lean body mass (SUL) was measured by manually drawing a volume of interest (VOI) around the target lesion(s). SULpeak was defined as the highest mean SUL in a 1.0-cm^3^ sphere voxel within the selected VOI and was determined automatically using a commercial software provided by scanner manufacture. Lesions with greater uptake than the reference threshold defined on the healthy liver or mediastinal blood pool without a pattern of inflammation or infection were defined as target lesion(s). A preset SUV cut-off window of 0–5 was defined for semi-quantitative and metabolic diameter measurements.

### Response criteria

A board-certified nuclear medicine physician reviewed the images while blinded to the patient’s previous [^18^F]FDG PET/CT reports and clinical features.

We applied three different, newly developed modified criteria: PERCIMT [13], PERCIST5 [22], and imPERCIST5 [20], to assess response to ICIs in this patient population (Table 1). Patients were classified into four groups based on treatment response on follow-up [^18^F]FDG PET/CT findings: complete metabolic response (CMR), partial metabolic response (PMR), progressive metabolic disease (PMD), and stable metabolic disease (SMD) according to PERCIMT, PERCIST5, and imPERCIST5 (Table 1).

**Table 1:** Definition of PET-based modified metabolic response to treatment criteria. *PET* positron emission tomography, *PERCIMT* PET Response Evaluation Criteria for Immunotherapy, *imPERCIST5* immunotherapy-modified PERCIST for up to Five Lesions, *PERCIST5* PERCIST up to 5 lesions

Responses	PERCIMT	PERCIST5	imPERCIST5
Progressive metabolic disease (PMD)	≥ 4 new lesions of less than 1 cm in functional diameter or ≥ 3 new lesions of more than 1.0 cm in functional diameter or ≥ 2 new lesions of more than 1.5 cm in functional diameter	Defined by an increase of the sum of SULpeak of the 5 lesions by 30% or appearance of new lesion(s)	Defined only by an increase of the sum of SULpeak of the 5 lesions by 30%
Stable metabolic disease (SMD)	Neither PMD nor PMR/CMR	Not meeting the definitions for CMR, PMR, or PMD	Not meeting the definitions for CMR, PMR, or PMD
Partial metabolic response (PMR)	Complete resolution of some pre-existing FDG-avid lesions. No new FDG-avid lesions	If the sum of SULpeak decreased by at least 30%	If the sum of SULpeak decreased by at least 30%
Complete metabolic response (CMR)	Complete resolution of all pre-existing FDG-avid lesions. No new FDG-avid lesions	Defined as the resolution of all malignant lesions and was nominally assigned as SULpeak of zero for quantitative analysis	Defined as the resolution of all malignant lesions and was nominally assigned as SULpeak of zero for quantitative analysis

For PERCIMT criteria, scans were reviewed with a focus on the appearance of new [^18^F]FDG-avid lesions, specifically in their absolute number and functional size (i.e., > 1.0 cm or > 1.5 cm). Functional size is the maximum metabolic diameter of pathologic lesion (in centimeters) measured on the fused PET/CT images [13]. According to PERCIMT, no new lesions and regression of all metabolically active lesions associated with melanoma were defined as CMR. PMR included no new lesions and remission of some FDG-avid lesions. PMD was defined when more than 4 new pathologic FDG-avid lesions, regardless of size, or 3 or more lesions greater than 1.0 cm or 2 or more lesions greater than 1.5 cm were detected. The remainder variations were classified as SMD when neither CMR/PMR nor PMD were seen (Table 1).

According to PERCIST5 criterion [22], the sum of SULpeak was measured in 5 metastatic lesions (no more than 2 per organ) with the highest SULpeak. The metastatic lesions could be at different locations on baseline and follow-up [^18^F]FDG PET/CT scans. CMR was defined as complete resolution of [^18^F]FDG uptake in the metastatic lesions without appearance of new metastasis on PET. PMR was defined as a decrease of more than 30% in the sum of SULpeak. PMD was defined as an increase of more than 30% in the sum of SULpeak in up to 5 prominent lesions or the appearance of new lesions. All other metabolic changes of [^18^F]FDG-avid lesions that did not fit into the definitions of CMR, PMR or PMD, were considered SMD.

Based on imPERCIST5, the reviewer determined the sum of SULpeak comprised no more than five lesions with the highest SULpeak. The difference in the sum of SULpeak was scored as (sum of baseline SULpeak minus the sum of follow-up SULpeak divided by the sum of baseline SULpeak multiplied by 100) [20]. Using imPERCIST5 criteria, CMR was defined when no new pathologic [^18^F]FDG-avid lesion was detectable, and all metastases disappeared. PMR included a decrease of more than 30% or an absolute rate of more than 0.8 in the sum of the SULpeak, regardless of a new lesion. PMD included an increase of more than 30% or more than 0.8 absolute rates in the sum of the SULpeak, independent of a new lesion. SMD is classified when the definitions for CMR, PMR, and PMD were not met (Table 1).

We calculated the “response rate” for each criterion separately. For this, the number of patients with CMR and PMR was divided by the number of all patients in the study and multiplied by 100. To assess the “disease control rate,” we also considered two groups based on each criterion: the “disease-controlled group” (CMR, PMR, and SMD) and the “uncontrolled-disease group” (PMD). A comparison between the three criteria was performed using these classifications. The predictive value of PERCIMT, PERCIST5, and imPERCIST5 criteria for PFS and OS was assessed. For this purpose, patients with CMR, PMR, and SMD were classified as “responders,” and cases with PMD were classified as “non-responders”.

### Statistical analysis

Data were checked for consistency and normality. Fisher’s exact test or Pearson’s test was used to analyze cross-tabulations. Generalized linear models with log-normal distributions were used for continuously distributed variables, and LSD tests were used for pairwise comparisons. McNemar’s test compared dependent proportions with 95% confidence intervals. Kaplan–Meier analyses were done using log-rank tests based on randomization tests. All reported tests were two-sided, and *p* values < 0.05 were considered statistically significant. All statistical analyses in this report were performed using NCSS (NCSS 10, NCSS, LLC, Kaysville, UT), STATISTICA 13 (Hill, T. & Lewicki, P. Statistics: Methods and Applications. StatSoft, Tulsa, OK).

## Results

### Patient characteristics and imaging

Overall, 91 patients with stage IV melanoma referred to our department between January 2015 and June 2017 were eligible to include in this study. The patient’s baseline characteristics are summarized in Table 2. Baseline scans were performed on an average of 28.3 ± 24.2 days before the ICI treatment session with a median interval of 20 days, and follow-up scans were performed on an average of 110.7 ± 35.5 days after the baseline [^18^F]FDG PET/CT with a median interval of 104 days. There were no therapeutic interventions for the patients between the baseline scans and the initiation of treatment and between the last treatment sessions and the follow-up scans. None of the baseline and follow-up scans was performed outside the recommended uptake time range (50–70 min after the injection). In all patients, the uptake time difference between the baseline and follow-up scans was less than 15 min. At the time of follow-up scans, patients had received a minimum of 2 and a maximum of 10 ICI doses. The number of doses received was unknown for eight patients at the follow-up scan. The last follow-up for calculating OS was performed on July 31, 2021. Overall, patients received a minimum of 2 and a maximum of 56 doses of ICIs at the end of the study (median of 9 doses).

**Table 2:** General characteristics of the patients, tumor, immunotherapy, and [^18^F]FDG PET/CT studies. *BRAF* B-Rapidly Accelerated Fibrosarcoma, *MEK* MAP/ERK kinase, *CMR* complete metabolic response, *PMR* partial metabolic response, *PMD* progressive metabolic disease, and *SMD* stable metabolic disease

Characteristics	Number	Percentage
Gender		
Female	36	36.9
Male	55	60.4
Melanoma site		
Cutaneous	78	85.7
Mucosal	5	5.5
Uveal	2	2.2
Unknown	6	6.6
Ulceration		
Yes	17	18.7
No	74	81.3
BRAF mutation		
Yes	30	33.0
No	61	67.0
Metastasis location		
Distant skin, subcutaneous or nodal metastases (M1a)	9	9.9
Lung metastases (M1b)	11	12.1
All other visceral metastases (M1c)	26	28.6
Brain metastases (M1d)	45	49.5
Type of regimen		
Nivolumab single therapy	28	30.8
Pembrolizumab single therapy	28	30.8
Ipilimumab + nivolumab	32	35.2
Ipilimumab + pembrolizumab	3	3.3
BRAF/MEK inhibitor	25	27
Reasons for treatment termination		
Complete response	14	15.4
Progression	50	54.9
irAE	7	7.7
Treatment completion	4	4.4
Patient request	2	2.2
Ongoing	9	9.9
Unknown	2	2.2
Clinical status at the end of follow-up		
CMR	28	30.8
PMD	46	50.5
SMD	10	11.0
PMR	5	5.5
Unknown	2	2.2

### Evaluation of response to treatment

Patients were divided into four groups: CMR, PMD, SMD, and PMR, based on each criterion. The CMR patients were the same in all three response criteria. The number and percentage of different classifications in the three approaches are presented in Table 3.

**Table 3:** Response to treatment according to PERCIMT, PERCIST5, and imPERCIST5. *CMR* complete metabolic response, *PMR* partial metabolic response, *PMD* progressive metabolic disease, and *SMD* stable metabolic disease

Response status	Number	Percentage (%)	Difference
PERCIMT (response-to-therapy)			
CMR	22	24.2	
PMD	26	28.6	
SMD	28	30.8	
PMR	15	16.5	
Response rate (CMR, PMR)	37	40.7	• PERCIMT versus PERCIST5: *P* = 1.0 • PERCIMT versus imPERCIST5: *P* = 0.0003 [95% CI for difference of proportions: 14.3% (8.5–22.9%)]
Responders (disease-controlled group: CMR, PMR, SMD)	65	71.4	• PERCIMT versus PERCIST5: *P* = 0.0004 [95% CI for difference of proportions: 20.8% (13.8–30.3%)] • PERCIMT versus imPERCIST5: *P* = 0.57
Non-responders (PMD)	26	28.6	
PERCIST5 (response-to-therapy)			
CMR	22	24.2	
PMD	45	49.4	
SMD	8	8.8	
PMR	16	17.6	
Response rate (CMR, PMR)	38	41.8	• PERCIST5 versus PERCIMT: *P* = 1.0 • PERCIST5 versus imPERCIST5: *P* = 0.0005 [95% CI for difference of proportions: 13.2% (7.7–21.7%)]
Responders (disease-controlled group: CMR, PMR, SMD)	46	50.5	• PERCIST5 versus PERCIMT: *P* = 0.0004 [95% CI for difference of proportions: 20.8% (13.8–30.3%)] • PERCIST5 versus imPERCIST5: *P* = 0.00001 [95% CI for difference of proportions: 24.2% (16.5–33.9%)]
Non-responders (PMD)	45	49.5	
imPERCIST5 (response-to-therapy)			
CMR	22	24.2	
PMD	23	25.3	
SMD	18	19.8	
PMR	28	30.8	
Response rate (CMR, PMR)	50	54.9	• imPERCIST5 versus PERCIMT: *P* = 0.0003 [95% CI for difference of proportions: 14.3% (8.5–22.9%)] • imPERCIST5 versus PERCIST5: *P* = 0.0005 [95% CI for difference of proportions: 13.2% (7.7–21.7%)]
Responders (disease-controlled group: CMR, PMR, SMD)	68	74.7	• imPERCIST5 versus PERCIMT: *P* = 0.57 • imPERCIST5 versus PERCIST5: *P* = 0.00001 [95% CI for difference of proportions: 24.2% (16.5–33.9%)]
Uncontrolled disease group	23	25.3	

**Fig. 2:**
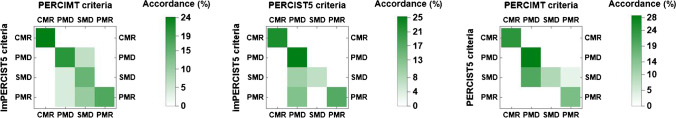
List density plots presenting an overview of accordance/discordance between different response criteria in patients with advanced melanoma. **a** imPERCIST5 versus PERCIMT. **b** imPERCIST5 vs. PERCIST5. **c** PERCIST5 versus PERCIMT. Green highlighted areas sum up to 100%. CMR = complete metabolic remission, PMD = progressive metabolic disease, SMD = stable metabolic disease, and PMR = partial metabolic response

Using the PERCIMT and PERCIST5 criteria, comparable results (*P* = 1.0) were found concerning the response rates (i.e., 40.7% and 41.8%, respectively), while it was significantly different (*P* < 0.001) from that of imPERCIST5 (i.e., 54.9%). PERCIMT and imPERCIST5 criteria showed disease control rates (i.e., CMR, PMR, SMD) of 71.4% and 74.7%, respectively (*P* = 0.57), which were significantly different (*P* < 0.001) from that of PERCIST5 criterion (i.e., 50.5%). The details of these findings are shown in Table 3. An overview of accordance/discordance between different response criteria is illustrated in Fig. 2.

The clinical status of the patients at the end of the follow-up period was also classified with a similar arrangement in four groups (CMR, PMD, SMD, and PMR). One of the 91 enrolled patients had unclear clinical status at the last follow-up. Overall, concordant findings were observed between modified response criteria and clinical status at the end of follow-up in 48% (43/90), 53% (48/90), and 41% (37/90) of patients by PERCIMT, PERCIST5, and imPERCIST5 criteria, respectively.

### Survival analysis

The median PFS time was 0.51 years (95% CI: 0.41–0.76). The median OS time was 2.01 years (95% CI: 1.87–2.51), and the mean OS was 2.19 ± 1.52 years. Among the type of ICI regimen and clinical risk factors, a meaningful relation was only reported for the previous history of chemotherapy with PFS (*P* = 0.001) and age ≥ 75 years with OS (*P* = 0.004). No significant correlation was found between BRAF mutation and a history of treatment with BRAF/MEK inhibitors and PFS or OS, despite the fact that 33% (30/92) of patients had BRAF mutation and 27% (25/92) of them were being treated with BRAF/MEK inhibitors. More details have been tabulated in Table 4.

**Table 4:** The effect of type of ICI regimen and clinical risk factor on patient outcome, PFS, and OS. *PFS* progression-free survival, *OS* overall survival, *BRAF* B-Rapidly Accelerated Fibrosarcoma, *MEK* MAP/ERK kinase, *ICIs* immune checkpoint inhibitors

Clinical risk factors	PFS	OS
Age at diagnosis ≥ 75 years	*P* = 0.40	*P* = 0.004
Gender	*P* = 0.42	*P* = 0.54
BRAF mutation	*P* = 0.14	*P* = 0.96
BRAF-MEK inhibitor therapy	*P* = 0.075	*P* = 0.76
Metastasis location	-	*P* = 0.14
Type of ICI regimen	-	*P* = 0.51
History of chemotherapy	*P* = 0.0012	*P* = 0.98

**Fig. 3:**
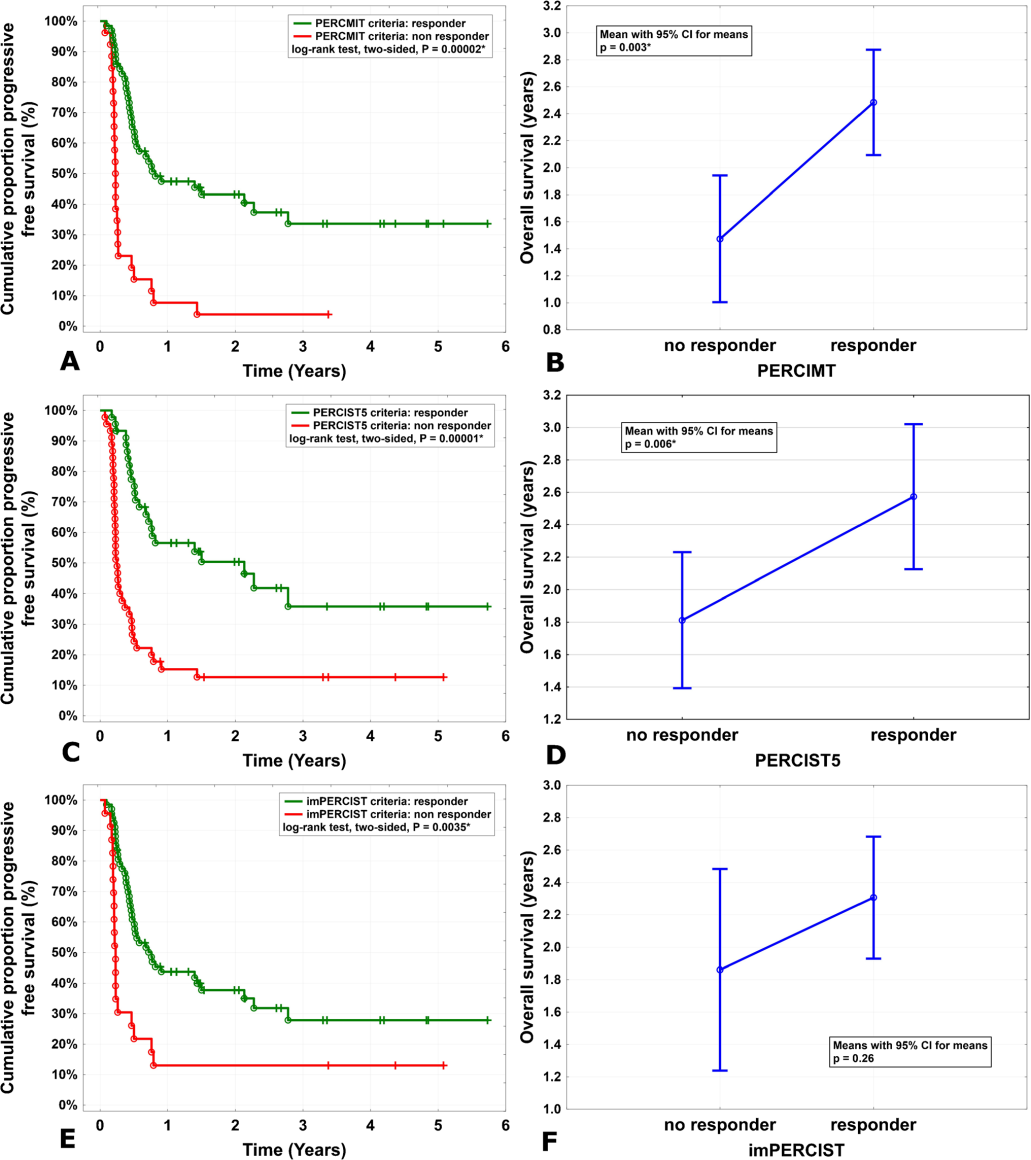
Comparison of the PFS (log-rank test) and OS (generalized linear model) in metabolic responder and non-responder groups based on PERCIMT (plans **A** and **B**), PERCIST5 (plans **C** and **D**), and imPERCIST5 (plans **E** and **F**) criteria

The PFS values showed a statistically significant difference between responders and non-responders based on all modified response criteria (*P* values: PERCIMT < 0.001, PERCIST5 < 0.001, and imPERCIST5 0.003), Fig. 3. Based on all three criteria, PFS was significantly longer in patients with CMR than in PMD (*P* < 0.001). The OS was also higher for CMR compared to PMD using PERCIMT and PERCIST5 criteria (*P* = 0.007 and *P* = 0.019, respectively). However, according to imPERCIST5, no significant difference in OS was observed between response groups (CMR, PMD, SMD, and PMR).

On the other hand, the OS was significantly longer in the metabolic responder groups as compared with the non-responder groups based on PERCIMT and PERCIST5 criteria (PERCIMT: 2.48 versus 1.47 years, *P* = 0.003; PERCIST5: 2.57 versus 1.81 years, *P* = 0.017). However, based on the imPERCIST5 criteria, this difference was not observed (*P* = 0.12). Table 5 shows the overall survival values for the different patient groups.

**Table 5:** The overall survival times in different response groups according to PERCIM, PERCIST5, and imPERCIST5. *CMR* complete metabolic response, *PMR* partial metabolic response, *PMD* progressive metabolic disease, and *SMD* stable metabolic disease

Response group	Number	Overall survival time
PERCIMT (response-to-therapy)		
CMR	21	2.66 ± 1.45
PMD	26	1.47 ± 1.16
SMD	28	2.53 ± 1.68
PMR	15	2.15 ± 1.56
PERCIST5 (response-to-therapy)		
CMR	21	2.66 ± 1.45
PMD	45	1.81 ± 1.43
SMD	8	2.80 ± 1.58
PMR	16	2.34 ± 1.68
imPERCIST5 (response-to-therapy)		
CMR	21	2.66 ± 1.45
PMD	23	1.86 ± 1.44
SMD	18	2.07 ± 1.51
PMR	28	2.19 ± 1.64
Overall survival in total	90	2.19 ± 1.52

## Discussion

The response patterns obtained after immunotherapy could be different from those observed after usual chemotherapy. From an imaging point of view, immunotherapy-modified criteria have been formulated to help handle these variegate patterns of response and to overcome the limitations of conventional criteria, both for anatomical and metabolic imaging. In the present study, we compared the efficacy of three metabolic response criteria, i.e., PERCIMT, PERCIST5, and imPERCIST5, to define their potentials and limitations.

**Fig. 4:**
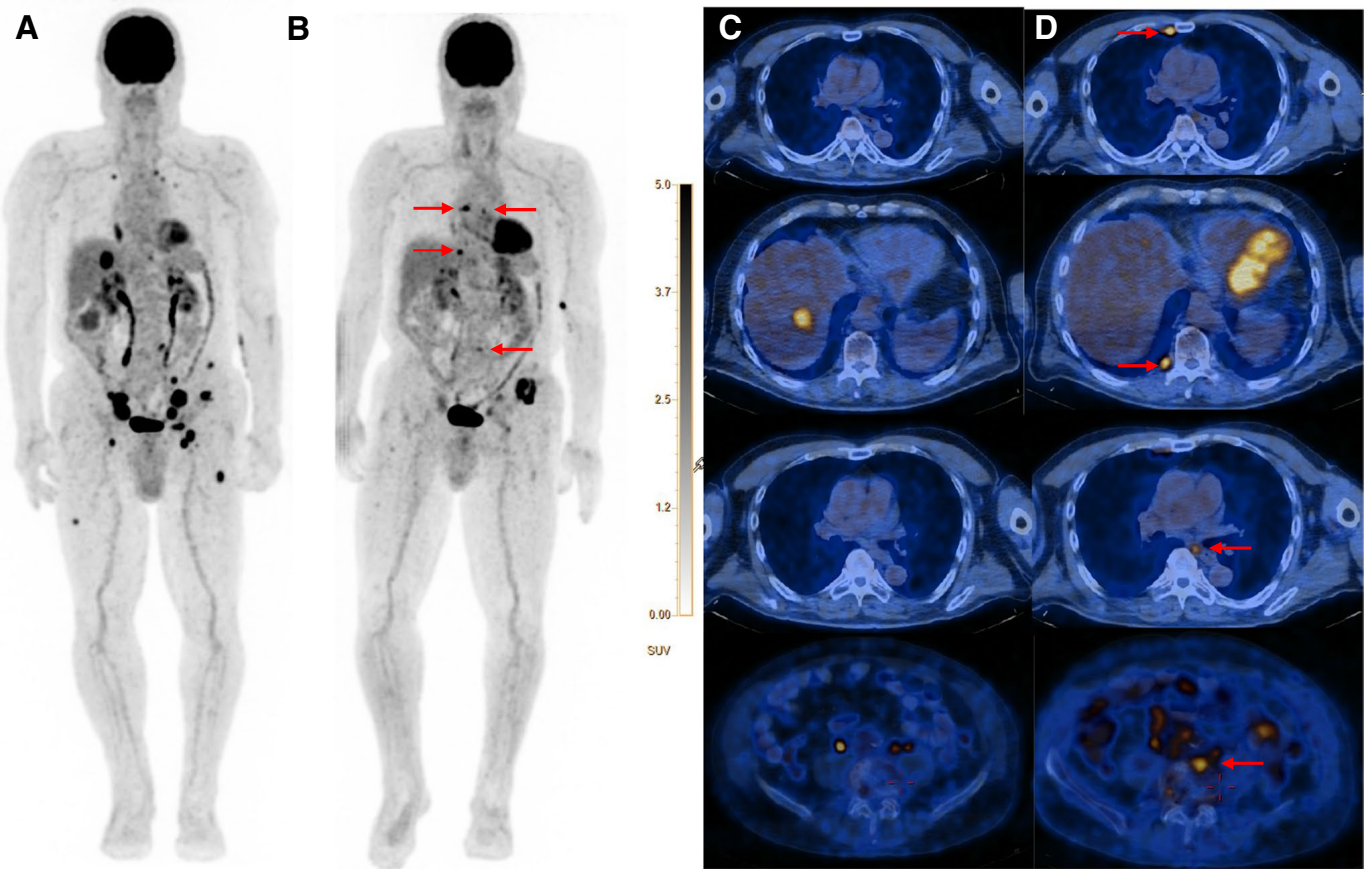
[^18^F]FDG PET/CT of a patient with advanced metastatic melanoma in staging (**A**, **C**) and treatment monitoring (**B**, **D**) 3 months after ICI treatment with nivolumab. [^18^F]FDG PET/CT before ICI treatment (**A**, **C**) shows multiple hypermetabolic pulmonal, hepatic, lymph node, and bone metastases, which were mainly improved after nivolumab treatment (**B**, **D**). In spite of metabolic response of most of primary metastases, at least 4 new lymph node and bone metastases were detected on 3-month follow-up [.^18^F]FDG PET/CT examination. The patient was classified as responder with PMR based on imPERCIST5, while he was non-responder with PMD based on PERCIMT and PERCIST5 criteria. Follow-up clinical and imaging studies showed progressive disease correlated with PERCIMT and PERCIST5

The distinction of pseudoprogression from true progression is one of the clinician’s challenges. We know that 40–45% of patients show no response to immunotherapy [1, 10]. On the other hand, the incidence of pseudoprogression does not exceed 10% [27]. Despite it seems the PERCIMT and PERCIST5 criteria compared to the imPERCIST5 have a more strict approach to classifying patients in PMD, a larger number of patients take place in the PMD based on PERCIMT and, particularly, PERCIST5 criteria. However, the significance of this difference was not assessed because of the small number of patients in each classification. Nevertheless, the significantly higher number of patients classified as having PMD according to PERCIST5 may pose a challenge for treatment decisions in routine clinical practice. On the survival analysis, the patients with PMD based on PERCIMT and PERCIST5 had significantly lower OS compared to the CMR and SMD group (Figs. 3 and 4), whereas the PMD patients according to the imPERCIST5 do not show a statistically meaningful difference in OS compared to other groups. In addition, the non-responder group of the PERCIMT and PERCIST5, which included only the PMD patients, had significantly lower OS compared to the responder group (i.e., CMR, PMR, and SMD), while this difference did not show between these two groups (responders versus non-responders) according to the imPERCIST5 (Fig. 3). In a way, considering the number and functional size of the new hypermetabolic lesion may be more efficient in the diagnosis of patients with PMD than a 30% increase in the sum of SULpeak of the 5 lesions. In the same way, Anwar et al. reported that a cut-off of four newly emerged hypermetabolic lesions based on the PERCIMT criteria has more predictive value for patient outcomes compared to the SUV changes [13]. Differently from our series, Annovazzi et al. reported the PERCIMT criteria had suboptimal performance in the identification of PMD patients (positive predictive value of 52.3%) [4]. Rivas et al. evaluated the metabolic response by PERCIST5 and imPERCIST5 criteria in patients with advanced or metastatic melanoma 3 months after first-line ICI therapy [22]. The authors found similar findings considering the predictive value of PERCIST5 and imPERCIST5 for patient’s outcomes. In contrast, in our study, using imPERCIST5 criterion, there was no significant difference in OS between responder and non-responder groups. In addition, a notably higher number of patients were classified as having PMD according to the PERCIST5 criterion. Ito et al. stated that modifications on the imPERCIST5 in the definition of PMD improved the diagnostic efficacy of the [^18^F]FDG PET/CT scan in response assessment of immunotherapy in melanoma [20]. Kitajima et al. demonstrated patients stratified by imPERCIST5 criteria in CMR and PMR had significantly longer OS in comparison with the PMD and SMD groups (*P* = 0.001) [28]. The diversity in the results of the latter studies may be related to the in homogenous patient cohorts with various ICI regimes and the duration of the treatment. In addition, they have evaluated either imPERCIST5 criteria or a combination of imPERCIST5 and metabolic PET parameters for assessment of clinical outcome and treatment response. However, to our best knowledge, this is the first study that compared the predictive value of three modified metabolic imaging criteria (PERCIMT, PERCIST5, and imPERCIST5) for clinical outcome in a relative homogenous patient’s population regarding clinical stage of the disease, ICI regimes, and the duration of the treatment.

Previous studies have reported response rates of 26–40% for different regimens of ICIs in melanoma patients [29, 30]. In the present work, we found response rates of 40.7–54.9% using three criteria, with comparable results between PERCIMT and PERCIST5, but significantly different to those of imPERCIST5 (Table 3). It should be noted that the type of ICI agents could be a source for this difference between the various studies. The accordance between response to treatment and clinical status at the end of the follow-up period based on the PERCIMT, PERCIST5, and imPERCIST5 was 48%, 53%, and 41% respectively. Response estimated by PERCIMT and PERCIST5 appears to be more consistent with clinical status at last follow-up.

One of our limitations was the relatively small population used to develop these criteria. It seems more comprehensive studies are needed for verification of the modified criteria. Additional larger studies with prospective designs can serve as a good guide to achieving consensus criteria for response assessment in melanoma patients treated with ICIs. It is also necessary to apply the modified criteria in standardization approaches of clinics. In addition, the PET/CT acquisitions were performed with two different scanners. This may result in non-identical SUV values between two scanner types. Furthermore, the recent EANM/SNMMI/ANZSNM joint practice guidelines for [^18^F]FDG PET/CT imaging in immunomodulatory treatment recommend the use of spleen-to-liver ratio as a sign of the systemic immune response [24]; however, this was not analyzed in this study. Finally, given the retrospective design of this study, we were not able to accurately assess all irAEs and correlate them with imaging and clinical findings. Therefore, it was not possible to make a reliable statement about the possible misclassification of irAEs by the modified response criteria.

## Conclusion

The results of this study indicate that although the appearance of new lesions may be secondary to an inflammatory response to ICIs and indicative of pseudoprogression, given the higher rate of true progression, the appearance of new lesions should be interpreted deliberately. Of the three evaluated modified criteria, PERCIMT appears to provide more reliable assessment of metabolic response that correlates strongly with overall patient survival.

## Supplementary Information

Below is the link to the electronic supplementary material.

Supplementary file1 (DOCX 12 kb)

## Data Availability

The detailed data generated during and/or analyzed during the current study are available from the corresponding author upon reasonable request.
