# Supplementary material for: Predictive value and accuracy of [18F]FDG PET/CT modified response criteria for checkpoint immunotherapy in patients with advanced melanoma
Source: Eur J Nucl Med Mol Imaging. 2023 May 4;50(9):2715–26. doi: 10.1007/s00259-023-06247-8 (PMC10317870; doi:10.1007/s00259-023-06247-8)
Supplement: Supplementary file 1 — Supplementary file1 (DOCX 12 kb) [file 259_2023_6247_MOESM1_ESM.docx]

| Nivolumab 3 mg/kg I.V. every two weeks until tumor progression |
| --- |
| Nivolumab 480 mg I.V. every four weeks (flat dose) until tumor progression |
| Pembrolizumab 2 mg/kg I.V. every three weeks until tumor progression |
| Pembrolizumab 400 mg I.V. every six weeks (flat dose) until tumor progression |
| Pembrolizumab 200 mg I.V. every three weeks (flat dose) until tumor progression |
| Ipilimumab 3 mg/kg I.V. plus nivolumab 1 mg/kg I.V. every three weeks for four cycles, continuation with 3 mg/kg nivolumab every two weeks until tumor progression |
| Ipilimumab 1 mg/kg I.V. plus nivolumab 3 mg/kg I.V. every three weeks for four cycles, continuation with 3 mg/kg nivolumab every two weeks until tumor progression |
| Pembrolizumab 2 mg/kg every three weeks for 24 months plus four doses of ipilimumab 1 mg/kg every three weeks |
| Pembrolizumab 200 mg every three weeks for 24 months plus four doses of ipilimumab 50 mg every six weeks |
| Pembrolizumab 200 mg every three weeks for 24 months plus four doses of ipilimumab 100 mg every 12 weeks |

**Supplement 1: Protocols for Immune checkpoint inhibitors (ICIs) treatment.**
